# Productivity and the pandemic: short-term disruptions and long-term implications: The impact of the COVID-19 pandemic on productivity dynamics by industry

**DOI:** 10.1007/s10368-021-00515-4

**Published:** 2021-09-14

**Authors:** Klaas de Vries, Abdul Erumban, Bart van Ark

**Affiliations:** 1 The Conference Board, Brussels, Belgium; 2 Faculty of Economics and Business, University of Groningen, Groningen, The Netherlands; 3 The Productivity Institute, University of Manchester, Manchester, UK

**Keywords:** Productivity, Pandemic, Labour reallocation, Digital transformation, Work-from-home, D24, O47

## Abstract

This paper analyses quarterly estimates of productivity growth at industry level for three advanced economies, France, the UK and the US, for 2020. We use detailed industry-level data to distinguish reallocations of working hours between industries from pure within-industry productivity gains or losses. We find that all three countries showed positive growth rates of aggregate output per hour in 2020 over 2019. However, after removing the effects from the reallocation of hours between low and high productivity industries, only the US still performed positively in terms of within-industry productivity growth. In contrast, the two European economies showed negative within-industry productivity growth rates in 2020. While above-average digital-intensive industries outperformed below-average ones in both France and the UK, the US showed higher productivity growth in both groups compared to the European countries. Industries with medium-intensive levels of shares of employees working from home prior to the pandemic made larger productivity gains in 2020 than industries with the highest pre-pandemic work-from-home shares. Overall, after taking into account the productivity collapse in the hospitality and culture sector during 2020, productivity growth shows no clear deviation from the slowing pre-pandemic productivity trend. Future trends in productivity growth will depend on whether the favourable productivity gains (or smaller losses) in industries with above-average digital intensity will outweigh negative effects from the pandemic, in particular scarring effects on labour markets and business dynamics.

## Introduction

In 2020, the COVID-19 pandemic dramatically disrupted people’s lives as well as their economic fortunes in the short-term with possible consequences for the long-term. The global economy experienced a recession of an unprecedented nature. According to The Conference Board, global GDP contracted by 3.7% and GDP per capita by 4.5% in 2020. This compares very unfavourable with the global financial crisis (GFC) when global GDP declined by less than 1% and per capita income by 1.8%.^1^ While one might expect significant growth rebound effects once the pandemic fades, it is unclear how the long-term growth rate of the economy will be affected.

The growth performance of advanced economies in 2020 was especially affected by the pandemic. GDP for the advanced economies fell by 4.7%, nearly 2% points more than the fall in the emerging markets, and GDP by per capita dropped by 5.2%. The larger decline in advanced economies can be partly explained by lockdowns and other government-mandated restrictions of mobility to mitigate the impact of the pandemic on people’s health which were not as much implemented in many lower-income economies. Moreover, the size of the services sector in the advanced economies is larger and has been more vulnerable to those restrictive measures.

The pandemic and subsequent government interventions in advanced economies have led to a seemingly perverse effect on productivity growth. Despite the dramatic drop in economic activity, labour productivity (measured as GDP per hour) in advanced economies increased by, on average, 1.1% in 2020, which is quite similar to the trend of the past decade. The reason is that according to The Conference Board’s series the decline in total hours worked (5.8%) was even bigger than the fall in real output (4.7%). Less than half of the decline in total hours (2.6 percentage point) was due to a drop in the number of persons employed while the remainder came from a fall in average hours per person employed. This decline in working hours has, to a large extent, resulted from business support programmes and furlough programmes for employees, which governments implemented to mitigate the short-term impact of the pandemic on business failures and employment. As a result, the average number of annual hours per worker in advanced economies dropped by more than 3% from 1718 to 1661 hours, though with large differences between countries and sectors.^2^

Productivity growth is, of course, best analysed in a long-term context (Krugman 1994).^3^ Investments in human and physical capital, technology and innovation are only materialising in improved business and economic efficiencies in the longer term. Short-term productivity changes during a recession, therefore, need to be carefully interpreted. For example:

- The numerator (output index) and denominator (input index) in the productivity equation can change rapidly and be highly volatile, which will exacerbate swings in the productivity index.
- Data revisions of value, prices and resulting volumes of output and inputs may be especially large during times of crisis due to distortions in data collection.
- Downward shocks in one period may create large rebound effects in subsequent periods and therefore obscure the underlying long-term drivers when looking at the short-term indicators on a quarter-by-quarter or month-by-month basis.
- A crisis may impact sectors in different ways causing large shifts in employment shares leading to temporary reallocations of labour between low productivity and high productivity sectors. This has especially occurred during the pandemic, as mobility restrictions and mandated business shutdowns disproportionally affected sectors that were highly dependent on direct customer-facing contacts, in particular hospitality services, the cultural sector and parts of the retail sector (except essential retail services, like supermarkets).
- During the pandemic, the utilisation of human capital has been affected as a result of fiscal support programmes for business and employment furlough programmes.
- The utilisation of physical capital, such as buildings and machinery and equipment, also declines rapidly during a crisis. Adjustments for capacity utilisation can be large, and highly different between industries.^4^

Despite those concerns, there has been much conversation and some hard evidence that apart from the crisis-related effects described above, the pandemic may also have caused genuine productivity improvements. Some of those pure productivity effects may be related to the accelerated adoption of digital technologies by businesses (Bloom et al. 2021a; Riom and Valero 2021; McCann and Vorley 2021). Other effects are due to a rise in the number of people working from home, which may have caused improvements in digital communications and an acceleration in the digital processing of business information (Barrero et al. 2020; Taneja et al. 2021). Some of those improvements may just cause transitory productivity effects, but others could be of a permanent nature (Bloom et al. 2021b; Bighelli et al. 2021; McKinsey 2021).

While it is too early to precisely determine what the long-term economic effects of the pandemic will be, this paper aims to examine the latest estimates of productivity by industry and the possible long-term implications for productivity growth. We analyse quarterly estimates of productivity growth for 36 industries in three advanced countries, France, the UK and the US, for 2020 and the first quarter of 2021. We remove the productivity effects from reallocations of working hours between industries to focus on the pure productivity gains or losses within industries in driving aggregate productivity growth.^5^

We find that while all three countries showed positive growth rates of aggregate output per hour in 2020 over 2019, after removing the effects from reallocations between low and high productivity industries, only the US showed positive productivity growth within its industries (1.5%), whereas the two European economies showed negative within-industry productivity growth rates (France at − 1.1% and the UK at − 1.9%).^6^

We then proceed by grouping the 36 industries using three taxonomies. The first taxonomy is a simple sector taxonomy based on type of activities, clustering industries in five main sectors: manufacturing, “other industry” (comprising agriculture, mining, utilities and construction), market services (excluding hospitality and culture), hospitality and culture, and non-market services. We find highly different within-industry productivity contributions between countries. For example, in the US, within-industry productivity contributions were broad-based. Manufacturing, other industry and market services (excl. hospitality and culture) all showed positive within-industry productivity contributions. In the UK, only manufacturing and other industry showed a modestly positive effect on aggregate productivity growth, whereas in France, non-market services were the only sectors with positive within-industry contributions to aggregate productivity growth.

Our second taxonomy considers one specific aspect of digital transformation during the pandemic, that is, the productivity effects from working from home (WFH). We find no evidence of within-industry productivity growth benefits for the top quartile of high-intensive WFH industries vis-à-vis medium-intensive ones (the two middle quartiles). In fact, as the medium-intensive WFH industries made bigger productivity gains, they may have been catching up with the high-intensive WFH industries by implementing the basics of WFH during the pandemic. It may take time and effort before the productivity effects from WFH, and especially the anticipated increase in hybrid WFH models combining part-time work-from-home and work-in-office models, will become large enough to show up in the data as a clear differentiator between strongly and weakly performing industries.

The third taxonomy, which looks at digital transformation more broadly, provides a somewhat more favourable perspective on the productivity impact of new digital technologies introduced during the pandemic. Distinguishing industries by their usage of digital technology, including industry purchases of ICT goods and services, the share of ICT specialists in total employment and the share of turnover from online sales, we found better long-term performance in above-average digital intensive industries during the pre-pandemic period (van Ark et al. 2019, 2021). During the pandemic, above average digital-intensive industries showed higher productivity growth than below-average ones. In the US, both groups performed about the same but better than in France and the UK.

Overall we conclude that, after adjusting for the large industry reallocation effects, and with the notable exception of the collapse in productivity in the hospitality and culture sector, the within-industry growth patterns during the pandemic showed no clear deviation from the slowing long-term productivity trend as established in our earlier work (van Ark et al. 2019, 2021).

This sobering conclusion implies by no means that the pandemic could not turn out to be a source of a potential sustained productivity improvement during the post-pandemic period. Our analysis suggests that digital transformation seems to have progressed during the pandemic through favourable productivity gains (or smaller losses) in industries that are above-average users of digital technologies. Productivity growth during the post-pandemic period will depend on whether such positive effects will outweigh possible negative effects from the pandemic, in particular scarring effects on labour markets and unfavourable business dynamics.

The large differences in productivity performance between countries during the pandemic also suggest that country-specific factors such as the response of innovation ecosystems to the opportunities for adoption of new technologies play an important role in the future. Such differences in policy environment may also have a significant impact on the within-country regional fortunes of a productivity revival.

The paper proceeds as follows. In Section 2 we discuss the aggregate trends in productivity growth in France, the UK and the US for 2020, and compare them with pre-pandemic performance. We briefly discuss some of the key data quality issues, and address the impact of the business support and furlough programmes on productivity. In Section 3 we outline the shift-share technique used to separate the pure or within-industry contributions to aggregate productivity growth from the industry reallocation effects. In Section 4 we present the results from our three taxonomies (type of activity, working-from-home and digital intensity). In the final section we conclude by outlining the implications of the pandemic for productivity growth in the long-term.

## Key aggregate productivity trends during the pandemic

### General overview up to the first quarter of 2021

**Fig. 1:**
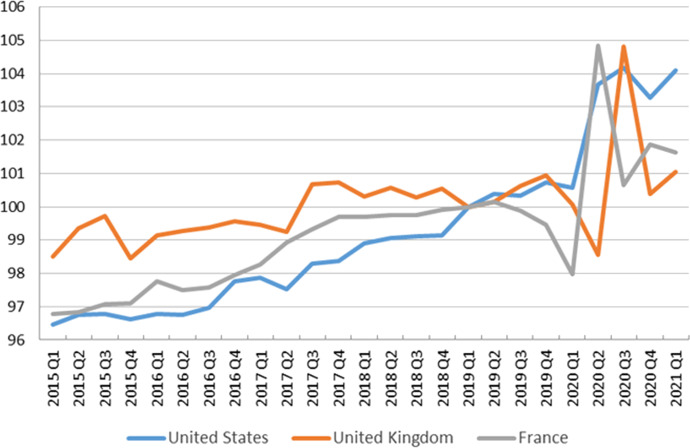
Real GDP per hour worked index (2019Q1 = 100), US, UK and France. *Sources* “Appendix 1”. INSEE (France), BEA and BLS (US), ONS (UK)

Over the course of 2020 and early 2021, labour productivity growth has been very volatile. Measured as GDP divided by total hours worked, it moved sharply up and down between quarters in France and the UK while it increased in the second quarter in the US without seeing any major downward correction since (Fig. 1; Table 1). On a yearly basis, productivity increased in all three countries because output declined less than the total number of hours worked. As explained below, these positive productivity effects resulted from active government interventions to mitigate the immediate economic fallout from the crisis even though the channels were quite different, especially between France and the UK on the one hand, and the US on the other.

**Table 1:** Growth rates of real GDP, total hours worked and labour productivity, 2020 annual average and 2020-Q1 to 2021-Q1 (q/q), France, UK and US (% change). *Sources* “Appendix 1”. Based on INSEE (France), BEA and BLS (US), ONS (UK)

	y/y (%)	Quarter over quarter change (%)				
	2020/2019	2020-Q1	2020-Q2	2020-Q3	2020-Q4	2021-Q1
France						
Real GDP	− 8.0	− 5.9	− 13.2	18.5	− 1.5	− 0.1
Total hours worked	− 9.2	− 4.4	− 18.9	23.4	− 2.7	0.1
Productivity	1.3	− 1.5	7.0	− 4.0	1.2	− 0.2
Employment rate (15–64)	66.1	66.8	65.2	65.9	66.5	66.5
Unemployment rate (15–64)	5.8	5.7	5.1	6.7	5.8	5.9
UK						
Real GDP	− 9.8	− 2.8	− 19.5	16.9	1.3	− 1.6
Total hours worked	− 10.3	− 2.0	− 18.2	10.0	5.7	− 2.2
Productivity	0.5	− 0.9	− 1.5	6.3	− 4.2	0.7
Employment rate (16–64)	75.4	76.3	75.7	75.0	74.7	74.7
Unemployment rate (16–64)	4.7	4.1	4.2	5.0	5.3	5.0
US						
Real GDP	− 3.5	− 1.3	− 9.0	7.5	1.1	1.6
Total hours worked	− 5.8	− 1.1	− 11.7	7.0	2.0	0.7
Productivity	2.5	− 0.2	3.1	0.5	− 0.9	0.8
Employment rate (16–64)	67.1	71.5	62.7	66.3	67.8	68.4
Unemployment rate (16–64)	8.2	3.9	13.1	8.8	7.1	6.1

The employment rate is calculated as a percent of the population; unemployment rate calculated as a percent of the labour force

**Fig. 2:**
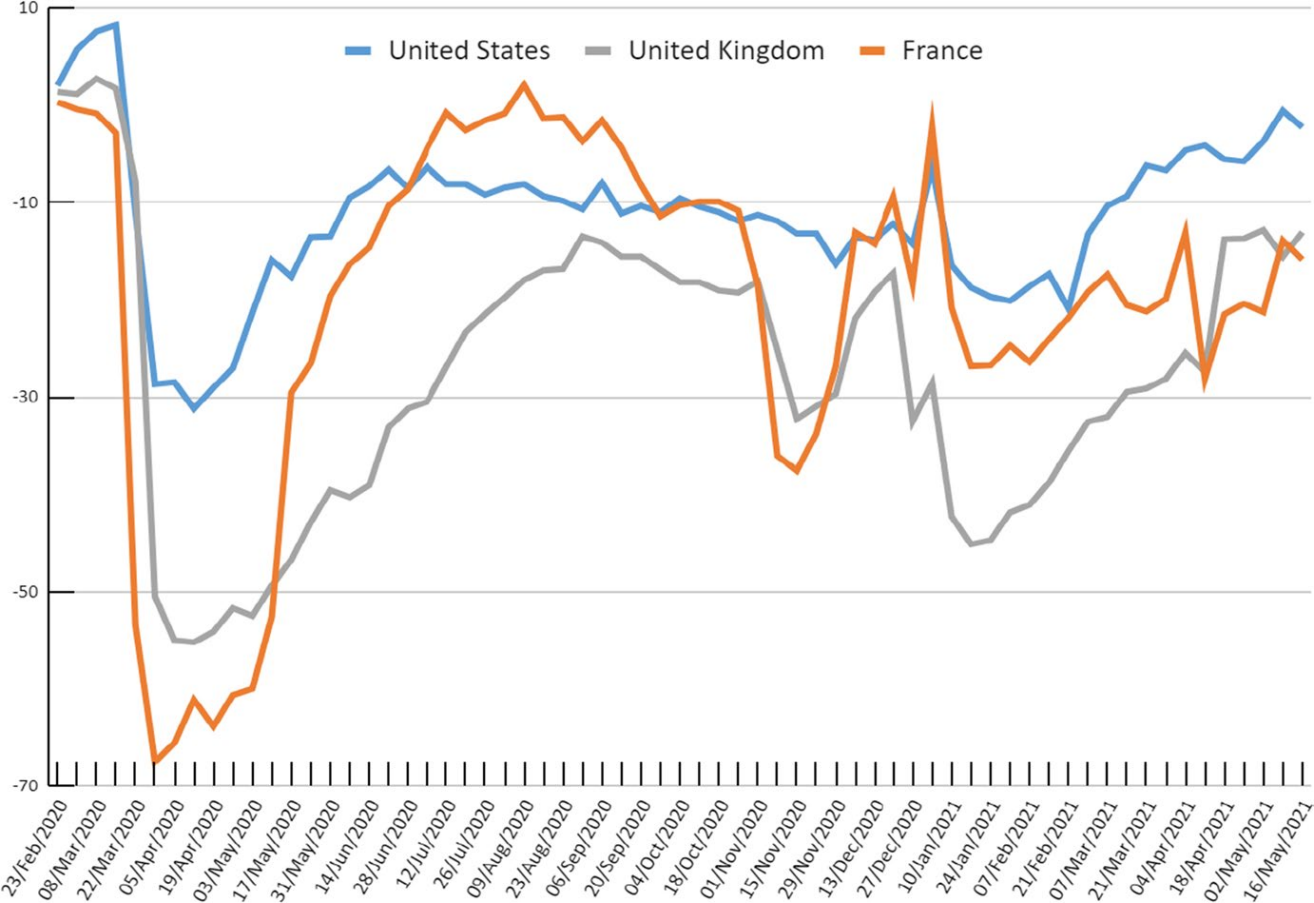
Mobility: weekly averages of trips to Grocery and Pharma and Retail and Recreation (% change from baseline), US, UK and France. *Source* own calculations using Google COVID-19 Community Mobility Reports, accessed on 27 May 2021

When the pandemic hit, all three economies recorded a sharp contraction in output in the second quarter of 2020 (Table 1). On March 11 2020, the World Health Organization declared COVID-19 a global pandemic, and by the end of that month many governments worldwide had implemented restrictions on the mobility of people, which caused a contraction of economic activity towards the end of Q1-20 and most of Q2-20. Along with generally increased uncertainty, this led to sharp drops in the mobility of persons though generally more so in France and the UK than in the US (Fig. 2). The fall in real output in Q2-20 was largest in the UK, while the US saw a much smaller drop in output because of smaller restrictions in mobility. In France and the US the impact of the pandemic on labour input, as measured by the total number of hours worked, was bigger than the reduction in output, leading to gains in measured labour productivity of 7.0% in the US and 3.1% in France in Q2-20. In the UK, hours fell slightly less than real output, so that productivity dropped by 1.5%.

By Q3-20, the three economies started to open up again as daily COVID-19 infections were significantly reduced. This led to a rebound in economic activity, thereby reversing many of the output and labour inputs losses induced in Q2-20. This was especially the case in France, where mobility indicators in Q3-20 came close to fully recovering to their pre-pandemic levels (Fig. 2), as did real GDP (Table 1). The recovery in labour input in France was even more impressive than the growth of output, resulting in a large drop in measured productivity—thereby erasing most of the productivity gains from Q2-20. In the UK, output also rebounded strongly, but total hours worked grew at a much more tepid pace, leading to a sharp increase labour productivity in Q3-20.

By Q4-20, daily infections were rising rapidly again as a second wave of COVID-19 cases got underway, leading to renewed lockdowns in France and the UK, though not as severely in the US. The economic impact of the renewed restrictions on mobility was much smaller compared to the first lockdowns in Q2-20. Restrictions were often more targeted towards specific economic activities and firms had learned to keep some business going despite the lockdowns. There was possibly also less fear among consumers to remain mobile as more information on the main transition mechanisms of the disease had become known.

In the UK, output increased slightly in Q4-20, while the labour market recovery was even much stronger. As a result, productivity growth recorded a sharp fall. In France, the renewed lockdown caused a fall in output though it was relatively small compared to the decline in Q2-20. In the US, growth in output was slower than the recovery in total hours worked, causing a modest decline in labour productivity for the first time since Q1-20.

On an annual basis, productivity levels in 2020 as measured by the aggregate data were slightly (in France and the UK) or substantially (in the US) above the pre-pandemic level of 2019. In Q1-21, when the third wave of COVID-19 hit, the UK saw a contraction in GDP with an even larger fall in hours worked, whereas the effects were more limited in France. In the US, output increased more than working hours during Q1-21, pointing at the beginnings of a pro-cyclical recovery path.

### The impact of business support and furlough programmes on productivity

In response to the pandemic, governments massively intervened to support businesses and workers during the crisis, but the effects on output, persons employed, hours worked and productivity were quite different between the three countries. In the US, unemployment increased rapidly, especially in sectors that were hardest hit by the pandemic, such as hospitality services and culture. Benefits were temporarily raised to cushion the blow for workers, but the link between employers and employees in lockdown industries was not retained. In France and the UK, this link between employers and employees was retained by sending workers home but continuing their pay on the basis of wage subsidies (or furlough schemes). The result of these divergent policies was a rapid rise in the unemployment rate in the US (from 3.9% in Q1-20 to 13.1% in Q2-20, and still at 6.1% by Q1-21) versus only small increases in the unemployment rates for France (from 5.7% in Q1-20 to 6.7% in Q3-20) and the UK (4.1% in Q1-20 to 5.3 in Q4-20). Meanwhile, all three countries provided direct financial support to businesses to remain afloat despite large income losses.

Because of those different schemes, if we would measure labour productivity as output per person employed instead of output per hour, the declines in labour productivity in the UK and France would have been much larger than an on output per hour-basis (as workers were still considered as in being employed). In contrast, output per person employed in the US it would have increased much more than output per hour (as workers were laid off). In terms of GDP per hour all three countries saw productivity go up, but more so in the US where output and total hours declined less than in France and the UK (see Table 1).

### Data quality issues

Due to the disruption of the pandemic, regular data collection in 2020 has also been hampered, leading to larger than usual uncertainties around the estimates of output and inputs (BLS 2021; ONS 2021a; OECD 2021a). The pandemic has also highlighted how differences in the measurement of volume estimates of GDP may impede international comparisons. For example, the UK’s Office for National Statistics highlights differences in the structure of the economy (e.g. the higher share of social consumption in the UK) and in measurement methods to explain the relatively large fall in UK's GDP compared to other G7 economies (ONS 2021a).

In particular, ONS argues that current price or nominal estimates should be more comparable on an international basis, and that the UK's performance based on that metric has not been all that different compared to other economies. Comparing a large set of economies, the OECD however finds that differences in government consumption and non-market output account for only a small part of cross-country variation in GDP growth (OECD 2021a).

**Fig. 3:**
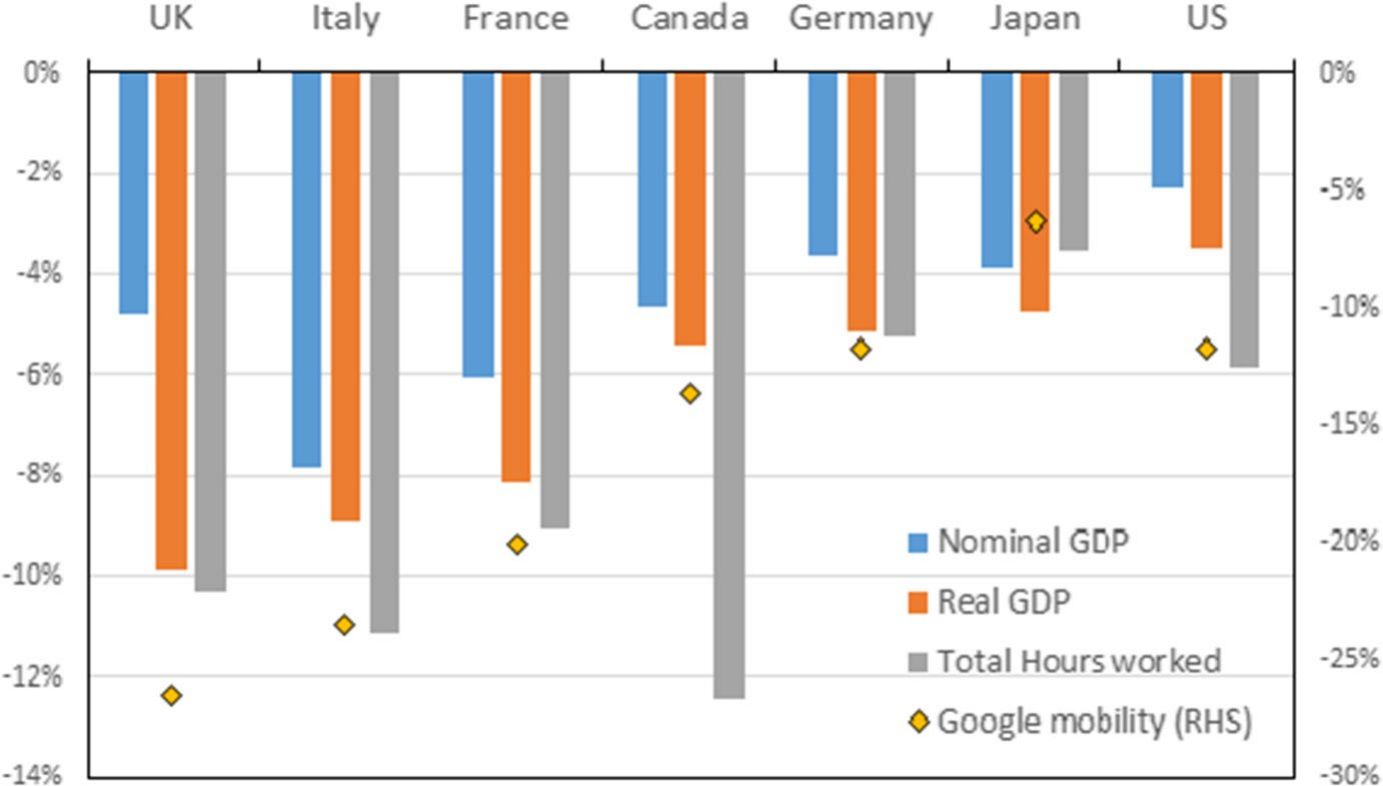
Growth rates of nominal and real GDP, total hours worked and Google mobility, G7, 2020 (% change). *Notes* Google mobility reports refer to the annual average of trips to Grocery and Pharma and Retail and Recreation as a percentage change from the baseline (the first 6 weeks of 2020). *Sources* own calculations using data from Google COVID-19 Community Mobility Reports, accessed on 27 May 2021; INSEE (France), BEA and BLS (US), ONS (UK); StatCan (Canada); Bbk (Germany); CAO and The Conference Board Total Economy Database (Japan)

Furthermore, alternative measures of economic activity, such as Google mobility measures, track the fall in real GDP in G7 economies (and in the case of the US, total hours worked) fairly well (Fig. 3). Figure 3 shows that the fall in total hours worked was also very similar to the fall in real GDP in most countries, with the notable exception of Canada. Both indicators (total hours worked and google mobility) dropped off much more in the UK than in most comparator countries. Hence these two data points support the view that the drop in real GDP in the UK was indeed among the largest in the G7.

Other issues related to measurement might involve the possible understatement of household output (including, for example, home schooling) and household inputs (including intangible assets in the household; see Eberly et al. 2021) during the lockdown, increases in hidden unemployment (less intensive work) and capacity utilisation adjustments. The latter especially complicates the measurement of total factor productivity, which we do not address in this paper except for the long-term projections in the final section. Finally, the pandemic also has led to a rethinking of the role of global supply chains in productivity growth, and a reassessment of how efficiency gains versus supply chain resiliency requirements affect the measures of productivity (OECD 2021b).

## Removing labor reallocation effects to measure pure industry productivity contributions

### The shift-share aggregation method

The aggregate productivity growth rates for 2020 as described in Section 2 have been highly impacted by large shifts of output and employment between industries. In particular, the measured reallocation effects (as discussed in this section) in Q2-20 and Q3-20 were abnormally high compared to any of the four quarters of 2019 or Q1-21 (see “Appendix 2” Table 7). Most notably the temporary closing and gradual reopening of firms in the hospitality and culture sectors, which are typically characterised by relatively low levels of labour productivity compared to sectors such as manufacturing or digital services, saw a large reduction in their share of output and employment causing positive reallocation effects on aggregate productivity growth in Q2-20 followed by partial rebounds in Q3-20 and Q4-20. In order to properly identify the within-industry effects, we employ the so-called shift-share approach in this paper.

There are different methods of aggregating industry-level productivity growth rates to distinguish between industry reallocation effects and pure within-industry effects on aggregate productivity growth. While these methods make relatively little difference in terms of empirical results during normal times, they do more so when output and labour input changes are volatile as was the case in 2020. A comparison of the shift-share approach used in this paper with two other alternative aggregation methods is provided in “Appendix 2”.

A common approach to measuring productivity growth is to assume an aggregate production function, which allows summing value added and hours across industries to obtain aggregate value added (Y) and aggregate hours worked (H) (see Jorgenson et al. 2012).^7^ With this additive property, one can measure aggregate labour productivity as the ratio of the Y and H:

1 $${y}_{t}=\frac{\sum {Y}_{i,t}}{\sum {H}_{i,t}}={Y}_{t}/{H}_{t},$$

where $${Y}_{i}$$ is the real value added and $${H}_{i}$$ is the number of hours worked, both for industry i. $$y$$ is the aggregate labour productivity measured as real value added per hour worked. This approach assumes identical industry value added functions so that aggregate GDP ($${Y}_{t})$$ is the sum of industry value added.

Using aggregate labour productivity as defined in (1), the shift-share decomposition approach separates the within-industry productivity effect from the labour input reallocation effects across industries (Fabricant 1942). In this approach, the absolute difference in aggregate productivity levels is decomposed into a pure within-industry productivity component, which is the change in industry productivity weighted by the relative employment size of the industry in the previous period, the change in employment share weighted by the productivity level in the previous period, and the product of changes in employment share and productivity level, i.e.

2 $${\Delta y}_{t}=\sum {{s}_{i,t-1}\cdot \Delta y}_{i,t}+\sum {{y}_{i,t-1}\cdot \Delta s}_{i,t}+\sum {{\Delta y}_{i,t}\cdot \Delta s}_{i,t},$$

where ${y}_{t},$ is as in (1), is the aggregate labour productivity obtained using aggregate production function, and ${s}_{i,t}$ is the employment share of the sector in the aggregate economy. Dividing both sides by the previous period aggregate labour productivity levels, we can express (2) in growth rate form as:

3 $$\frac{{\Delta y}_{t}}{{y}_{t-1}}={\dot{y}}_{t}=\frac{\sum {{s}_{i,t-1}\cdot \Delta y}_{i,t}}{{y}_{t-1}}+\frac{\sum {{y}_{i,t-1}\cdot \Delta s}_{i,t}}{{y}_{t-1}}+\frac{\sum {{\Delta y}_{i,t}\cdot \Delta s}_{i,t}}{{y}_{t-1}},$$

where ${\dot{y}}_{t}$ is the growth rate of aggregate labour productivity. The first term is the absolute change in each industry's productivity level relative to the aggregate economy productivity, weighted by the previous period's employment shares. This will be positive if the industry productivity improves, and the magnitude of the positive value depends upon the relative size of the industry in terms of employment. In other words, when the productivity of an industry improves, the aggregate productivity also improves in proportion to the industry size in terms of employment. This is the “pure” or within-industry productivity effect, which is the focus of this paper.

The second and last terms are the static and dynamic worker reallocation—or workers' movement from low productivity to high productivity sectors, which together make up the reallocation effect. The static shift-effect measures the relative level of industry productivity weighted by the absolute change in employment share from the previous period. This effect will be negative if employment expands in sectors with relatively lower productivity levels. The dynamic shift effect represents the change in industry productivity relative to aggregate productivity in the previous period and the change in employment share. Therefore, when employment expands in sectors where productivity growth is faster, it adds to aggregate productivity growth. The static reallocation captures the aggregate productivity effect of employment expansion in sectors with a relatively higher level of productivity, whereas the dynamic reallocation captures the effect of employment expansion in fast-growing sectors.

While decomposing aggregate productivity growth using Eq. (3), we use detailed industry data on output and labour input—66 industries for the UK, 50 for France, and 48 for the US. The level of industry details impacts the magnitude of reallocation effects, and the contribution of pure productivity effects. At a higher level of industry grouping, the potential to pick up the effects of movements of output or employment across industries is less compared to a lower level of industry groupings. For instance, if we take three sectors, such as agriculture, industry, and services, then the decomposition method only captures labour input movements between these broadly defined sectors. In contrast, if we have detailed industries within these three broad groups, the decomposition captures all the movements even within these broad sectors.^8^

## Within-industry productivity effects on basis of industry taxonomies

### Description of the taxonomies

In order to detect patterns in the productivity data over the course of the recession and recovery, we applied the shift-share method described in Section 3 to the available industry data which we subsequently grouped into 36 industries using three taxonomies (Exhibit 1). The first taxonomy clusters industries in similar types of activities, such as physical production (manufacturing and non-manufacturing), and private (market) and public (non-market) services delivery. The “other industry” group includes activities such as agriculture, mining, utilities and construction. Hospitality and culture, which includes hotels, restaurants, arts, entertainment and recreation (ISIC Rev.4 codes I55-56 and R90-93), are grouped together as they were most impacted by government-mandated restrictions over the course of the pandemic. Non-market services mainly include government, education, human health and social care activities. It should be noted that in the US a large part of non-market services is carried out by the private sector business, limiting the comparability of non-market services between countries somewhat. We removed a large part of the real estate industry from our analysis representing owner-occupied housing which is unrelated to any specific workforce activity (see “Appendix 1”).

**Exhibit 1:** Taxonomies based on type of activity, working-from-home and digital intensity

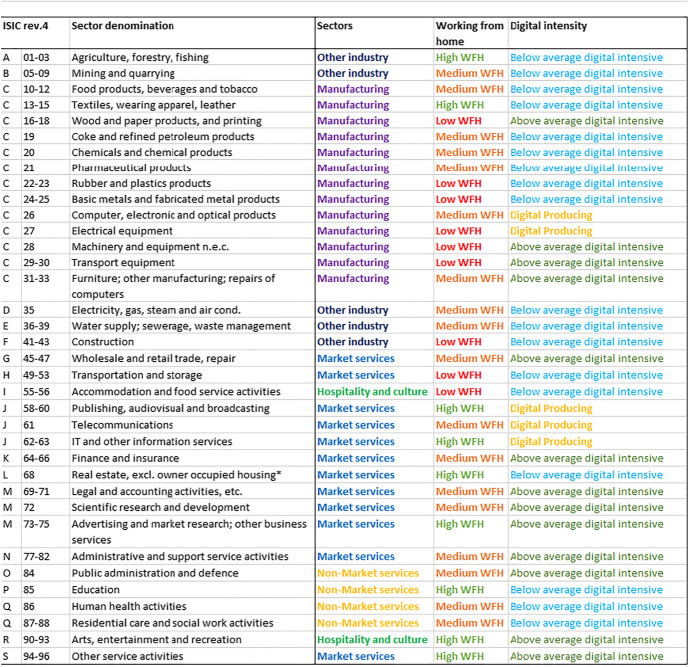



Note:* Excludes output computations for owner-occupied housing (see Appendix A).

The second taxonomy allows us to look at the productivity impact of working-from-home (WFH) during the pandemic. We used detailed data from the American Time Use Survey (ATUS), and averaged the values of the prevalence of work-from-home by industry over the period 2011–2019. To convert the ATUS worker level data, which are based on occupational classification from the US Census, to our industry classification, we relied on the underlying crosswalks and codes from Hensvik et al. (2020). Similar to their approach, we aggregated the share of WFH hours by industry from worker-level data to create our taxonomy. We identify three groups of industries, based on a quartile distribution with the two middle quartiles qualifying as ‘medium working-from-home’. Two important assumptions, guided by the availability of data, are made when applying this taxonomy to the three countries in this paper. The first is that we assume that WFH patterns by occupation/industry in the US are not very dissimilar from those in the UK and France. The second is that we use historical data to determine the prevalence of working-from-home by industry, although we acknowledge that most industries will have increased WFH activities in 2020 (ONS 2021c).

The third taxonomy looks at the digital intensity of sectors, following our earlier work on digital transformation and productivity growth (van Ark et al. 2019, 2021). For this, we adopted the digital intensity taxonomy developed by the OECD, which uses multiple dimensions of digital transformation related to technology, market and human capital-related features (Calvino et al. 2018). These include the share of intermediate purchases of ICT goods and services, the share of ICT specialists in total employment and the share of turnover from online sales. Using an overall summary indicator (the ‘global taxonomy’), we collapsed industries at the ISIC Rev.4 level into two groups: above average and below average digital-intensive industries. We also separated out a third group of industries which are producing digital goods and services, including computers, electronic and electrical equipment, and telecom and other digital service. Hence our above and below average digital-intensive industries are essentially digital ‘using’ industries.

### Sector taxonomy results

The first two columns of Table 2 provide an overview of the shares of each sector in nominal value added and total hours worked. The last column provides the level of productivity in each sector relative to that of the aggregate economy in 2019. In contrast to common wisdom, we find no significant difference in the size of the manufacturing sector between the three countries. The biggest difference between the three countries is that the size of the market services sector is slightly bigger in the UK than in France, whereas non-market services are bigger in France than in the UK.^9^ We also find, as expected, that productivity levels in manufacturing and market services (excl. hospitality and culture) are higher than those of other sectors in all three economies. Shifts between those sectors therefore drive some of the large industry reallocation effects in 2020, as described above.

**Table 2:** Pre-pandemic output and hours shares and productivity levels using taxonomies (2019). *Sources* “Appendix 1”. Based on INSEE (France), BEA and BLS (US), ONS (UK)

	Nominal value added share (%)			Hours worked share (%)			Productivity level (total economy = 1.00)		
	France	UK	US	France	UK	US	France	UK	US
Sectors									
Manufacturing	12	11	11	10	9	9	1.25	1.20	1.24
Other industry^a^	11	12	9	12	11	10	0.81	1.07	0.84
Market services^b^	50	52	48	46	49	44	1.11	1.02	1.10
Hospitality and culture	5	5	4	7	8	8	0.65	0.53	0.54
Non-market services	23	20	28	26	23	29	0.89	0.98	0.96
Work-from-home (WFH) intensity									
High WFH	24	24	18	20	21	19	1.20	1.11	0.96
Medium WFH	56	56	66	58	54	58	0.99	1.03	1.13
Low WFH	19	20	16	22	24	23	0.85	0.81	0.71
Digital intensity									
Above average digital-intensive^c^	45	47	58	49	48	54	0.94	0.95	1.07
Below average digital-intensive	48	45	33	47	47	42	1.00	0.99	0.79
Digital producing	7	9	9	4	6	4	1.74	1.49	2.07

^a^Other industry includes agriculture, mining, utilities and construction

^b^Market services excludes hospitality and culture as well as owner occupied dwellings

^c^Excluding digital producing industries; productivity levels are calculated on the basis of nominal value added data

Focusing on the contributions to productivity growth from industries (excluding the reallocation effects) in 2020, we find some important differences between the three countries (Table 3). In the UK, the manufacturing sector and the “other industry” sector contributed positively to within-industry productivity growth, whereas the three service sectors performed negatively, in particular non-market services (and especially education and health care industries). In France, manufacturing, other industry and hospitality and culture showed negative within-industry productivity contributions, whereas market services and non-market services performed positively. In the US, the manufacturing sector (in particular industries producing primary metals), other industry (in particular oil and gas exploitation and construction) and many market service activities (with air transportation services being the main exception) showed positive within-industry productivity growth rates in 2020. As in the two European countries hospitality and culture and non-market services (in particular the healthcare industry) showed negative within-industry productivity growth rates in the US.

**Table 3:** Decomposition of within-industry productivity effects using three taxonomies, annual average (% change). *Sources* “Appendix 1”. Based on INSEE (France), BEA and BLS (US), ONS (UK)

	France			UK			US		
	1998–2006	2011–2019	2020	1998–2006	2011–2019	2020	1998–2006	2011–2019	2020
Aggregate output per hour	1.5	0.9	0.9	1.9	0.3	0.2	2.0	0.8	2.3
Within-industry productivity	1.4	0.8	− 0.6	2.1	0.5	− 1.9	2.2	1.1	1.5
Static effect	0.2	0.1	1.4	− 0.1	− 0.1	2.2	− 0.1	− 0.3	0.8
Dynamic effect	− 0.1	0.0	0.1	− 0.1	− 0.2	− 0.1	− 0.1	− 0.1	0.0
Sectors									
Total within-industry effect	1.4	0.8	− 0.6	2.1	0.5	− 1.9	2.2	1.1	1.5
Manufacturing	0.5	0.2	− 0.3	0.6	0.0	0.2	0.6	0.1	0.6
Other industry^a^	0.2	0.0	− 0.5	0.2	0.0	0.1	0.0	0.2	0.5
Market services^b^	0.5	0.4	0.4	1.2	0.5	− 0.5	1.2	0.7	0.7
Hospitality and culture	0.0	0.0	− 0.3	0.1	− 0.1	− 0.5	0.1	0.0	− 0.3
Non-market services	0.2	0.2	0.2	0.0	0.1	− 1.2	0.3	0.1	0.0
Work-from-home (WFH) intensity									
Total within-industry effect	1.4	0.8	− 0.6	2.1	0.5	− 1.9	2.2	1.1	1.5
High WFH	0.2	0.2	0.5	0.1	0.2	− 0.9	0.3	0.3	0.5
Medium WFH	0.9	0.6	0.7	1.5	0.4	− 0.5	1.6	0.8	0.9
Low WFH	0.3	0.1	− 1.8	0.5	0.0	− 0.4	0.3	0.0	0.0
Digital intensity									
Total within-industry effect	1.4	0.8	− 0.6	2.1	0.5	− 1.9	2.2	1.1	1.5
Above average digital-intensive^c^	0.6	0.4	0.1	1.0	0.5	− 0.3	1.2	0.5	0.4
Below average digital-intensive	0.6	0.3	− 1.0	0.7	− 0.1	− 1.5	0.6	0.2	0.5
Digital Producing	0.2	0.2	0.3	0.4	0.1	− 0.1	0.4	0.5	0.6

^a^Other industry includes agriculture, mining, utilities and construction

^b^Market services excludes hospitality and culture as well as owner occupied dwellings

^c^Excluding digital producing industries

We conclude that the gain in productivity during the pandemic was broad-based in the US, and that any drop in output was more than offset by a drop in working hours for most US industries. In contrast, the two European countries retained a fair amount of less productive hours despite the extensive use of furlough programmes pointing at an underutilisation of labour. What this better US performance means for the recovery potential coming out of the pandemic, requires a closer look at the productivity contributions according to the other two industry taxonomies.

### Working-from-home taxonomy results

There has been much discussion to what extent the rapid acceleration in working-from-home has driven productivity improvements. Barrero et al. (2020) observe that the number of full workdays from home in the US increased from 5% in the pre-pandemic period to 20% during the pandemic. The Office of National Statistics in the UK reported that, during the second wave of COVID-19 infections in February 2021, 37% of persons employed worked fully from home, 10% worked partly from home and partly from work, and 34% travelled into work permanently. By the third week of June 2021, those travelling into work permanently had gone up to from 34 to 49%, 15% of persons employed were on a hybrid model, and only 22% worked from home entirely (ONS 2021e). So as the share of workers from home drops as economies emerge from lockdowns and mobility restrictions get eased, a fair amount of WFH is likely to remain because it has worked well for many employers and workers. One UK survey found that as much as 40% of workers would like to have two to three workdays from home by 2022, with the remainder equally split between 0–1 and 4–5 days (Taneja et al., 2021).

Barrero et al. (2021) suggest a 5% boost in US productivity in the post-pandemic period because of re-optimised working arrangements. The Conference Board (2021) is more cautious in predicting permanent productivity gains from WFH. Its survey data from US employers during the fall of 2020, suggest that much of the output gains may have resulted from longer working hours rather than higher productivity from home workers. The study also warns of the potential negative impact of WFH on collaboration and organisational culture.

The results from our industry taxonomy confirm the caution on the productivity effects from WFH as we see no clear advantage from within-industry productivity contributions from industries that showed the highest pre-pandemic WFH prevalence. In the UK the high-intensity WFH industries even contributed negatively to productivity growth, in particular due to negative contributions from the education industry. In the US, the within-productivity contribution from high-intensity WFH industries was moderately positive (0.5%) but less than the contribution of medium-intensity WFH industries (0.9%). Clearly, low-intensity WFH industries performed worse in all three countries, but for many industries in that group, the potential for working-from-home is much lower because of either the production-oriented or customer-facing nature of the business.

We conclude that, despite the rapid rise in WFH during 2020, the productivity effects are not clearly visible yet, especially not in industries where the WFH intensity was the highest in the pre-pandemic period. These results align with Wang (2021), who also finds no correlation between WFH intensity and productivity growth in Canada in 2020. However, as the taxonomy is based on pre-pandemic data, industries which were classified as medium-intensive WFH during the pre-pandemic period may have benefited in terms of productivity growth terms as they moved towards higher-intensity WFH practices during 2020. Those productivity effects may reflect low hanging fruit from more efficient communications and time savings from commuting. It may still take time and considerable effort before WFH will generate sustained improvements in productivity growth through new working processes which need to align with a continuous evolution of digital architectures, raising the need for better digital skills, safer data security protocols, etc.

### Digital usage taxonomy results

Positive productivity effects from the pandemic are more clearly visible from digital transformation in a broader sense. In our earlier work, we documented improvements in productivity growth in above average digital-intensity industries for most of the decade between the Global Financial Crisis and the COVID-19 pandemic, despite slowing aggregate productivity growth during that period especially since 2011 (Table 3). The Euro Area and the UK showed larger productivity contributions from above average digital-intensive industries, especially after 2013. And even though US productivity in the past decade was mostly driven by high productivity in digital producing industries, above-average digital-intensive industries outperformed the least intensive ones by a wide margin. Examples of strong productivity growth in intensive digital-using industries include many services industries, such as finance, trade and business services (van Ark et al. 2019, 2021).

By applying the digital usage taxonomy to data for 2020 we find a continuation of the gradual strengthening impact of digital intensity on productivity. The productivity contributions from the above average digital-intensive industries in the UK turned negative in 2020, and it was barely positive for France. However, in both countries this group of industries outperformed below average digital-intensive industries by a much wider margin than before the pandemic. The gap in the productivity contributions between the two sectors was 1.1 percentage points in France and 1.2 percentage points in 2020 compared to much smaller gaps from 2011 to 2019 (Table 3). For the US, the productivity contribution of both the above-average and below-average digital-intensive industries is somewhat comparable at 0.4 to 0.5 percentage points of the 1.5% within-industry productivity growth. The remaining 0.6% originated from the relatively large digital producing sector which (in 2019) accounted for only 9% of the US value added and 4% of total hours worked.

Overall, there are reasons to be optimistic about productivity gains from digital transformation during the post-pandemic era, especially in industries which already showed above average digital-intensity before the pandemic. Firms which showed a good record of technology adoption in the past are usually better in continuing to do so. For below average digital-intensity industries the potential for catching up could be substantial, especially because of a strengthening in technology adoption and the introduction of new management practices during the pandemic (Riom and Valero 2021; McKinsey 2021). However, the process of digital transformation is a lengthy one. The time lag between adopting the new technologies and the time by which they show up in productivity are related to learning effects, giving an advantage to industries which had already realised those effects earlier. There are also a substantial number of firms at risk of falling behind in their digital transformation process which may either fail in due course or may only survive in a less competitive environment in which productivity is not necessarily a growth driver.

## Conclusions and productivity outlook

The dynamics of productivity growth are best understood in the longer term. However, in times of crisis, analysing short-term productivity trends can help to reflect on whether the crisis might cause lasting damage to productivity or create opportunities for a revival in productivity growth. After removing significant industry reallocation effects, this paper analyses the pure within-industry contributions to aggregate productivity growth in 2020. Using various taxonomies, the underlying within-industry contributions to productivity growth provide useful information on the extent to which the pandemic may have weakened or strengthened drivers of productivity growth in the longer term.

Our results indicate that digital transformation during the pandemic seems to have progressed in industries with above-average digital intensity. We also find that the productivity performance in medium-intensive work-from-home industries is better than in high-intensive ones, suggesting that the former may be showing signs of catching up with the latter. Yet, after taking account of the productivity impact of the collapse in the hospitality and culture sectors, the remaining within-industry productivity growth patterns during 2020 do not exhibit a clear deviation from the slowing long-term trend productivity trend established in our earlier work (van Ark et al. 2019, 2021).

We find important differences in 2020 productivity growth between countries, especially between France and the UK on the one hand, and the US on the other. Beyond hospitality and culture, the stronger within-industry productivity contributions in the US may simply result from the heavy shedding of persons and total working hours at the start of the pandemic. In contrast, the European furlough programmes may have caused companies to keep more hours on the payroll than they would have done otherwise given the collapse in output. While the average unemployment rate in the US in Q1-21 was still slightly higher than in the UK and France, its employment rate was slightly above that of France but well below that in the UK (see Table 1). It remains to be seen whether a further recovery in the US labour market upholds faster productivity growth compared to France and the UK, or whether an increase in the employment rate slows the pace of within-industry productivity gains.

**Fig. 4:**
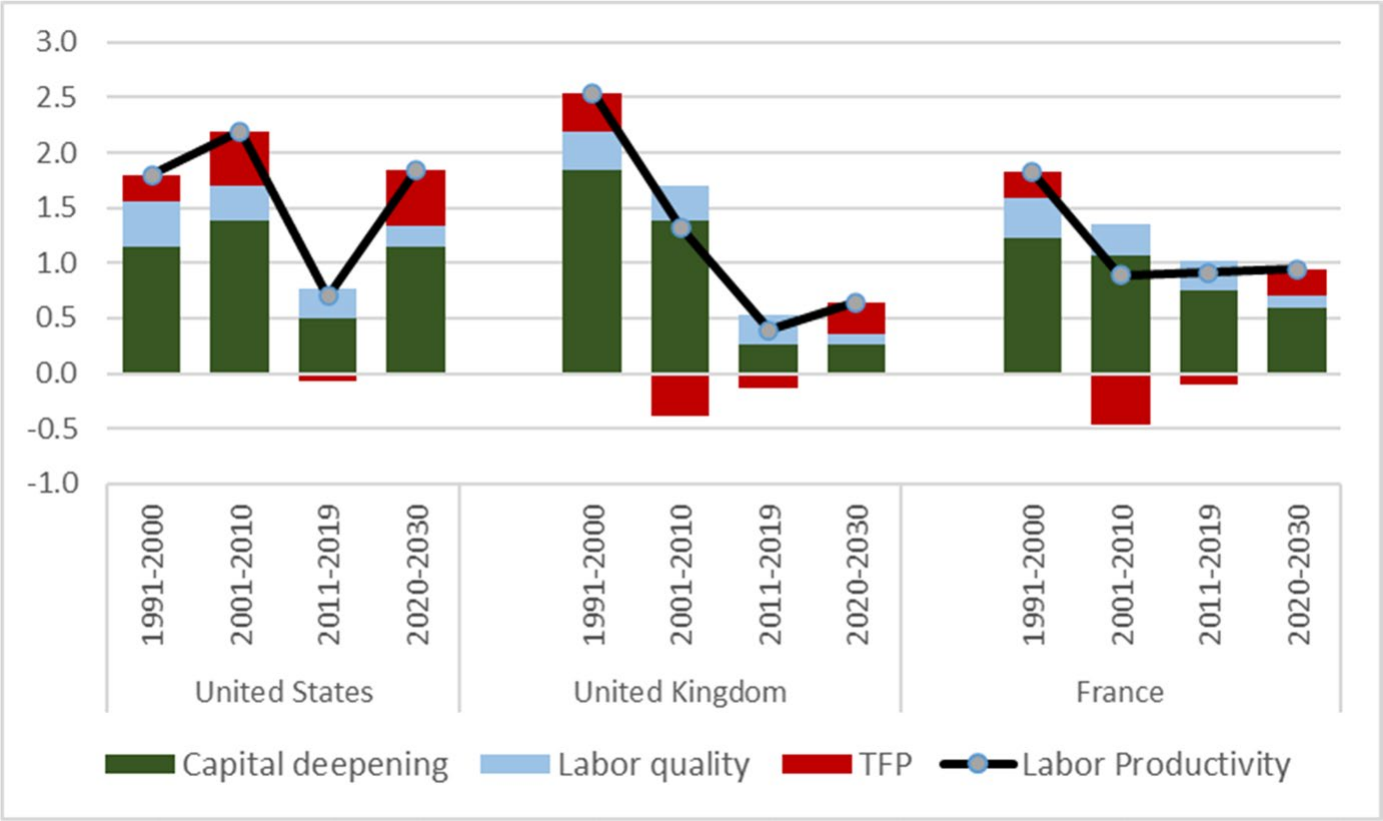
Contribution of factor deepening, factor quality and efficiency changes to labour productivity growth. *Source* The Conference Board Global Economic Outlook, 2021. For methodology see Footnote 10 and Erumban and de Vries (2018)

Projections by The Conference Board point to a stronger recovery of the long-term trend in productivity growth in the US than in France and the UK (Fig. 4). The projections are based on period-average projections of the contributions of capital deepening (measured as capital services per hour worked), labour quality (measured by educational attainment levels of the workforce) and total factor productivity.^10^ The projections suggest that US labour productivity growth could see a recovery from 0.7% in the past decade (2011–2019) to 1.8% in the next decade (2020–2030), which is comparable to the average productivity growth rate in the US from 1990 to 2010. In contrast, the projections for the two European countries suggest productivity growth rates much closer to the lower rate of the past decade (2010–2019), namely 0.9% in France and a slight increase from 0.4% (2011–2019) to 0.6% (2020–2030) in the UK. A significant pickup in capital deepening is the main driver of faster labour productivity growth in the US, whereas capital deepening remains largely unchanged in France and the UK. All three countries see a modest return to positive total factor productivity (TPF) growth during the next decade. Overall the results suggest that digital transformation, at least in the US, remains biased towards the growth of capital and total factor productivity. This appears to support the arguments of Acemoglu and Restrepo (2019) that the displacement effects in the labour market (shifting the task content of production against labour) are stronger than the reinstatement effects (because of the emergence of new production tasks in favour of labour).^11^

With the pandemic in the rearview mirror, how can it create any upside opportunity regarding a rise in investment and TFP growth in the coming decade? This paper identified various key factors which have emerged during the pandemic, including an acceleration in digital transformation and a productivity catch-up in medium work-from-home industries. There will also be an important role for human capital or increased labour quality, as identified in Fig. 4. Estimates of labour quality growth in the UK, measuring the mix of skills held by workers employed, showed a positive contribution to output growth during the pandemic, suggesting that the highest skilled workers were able to continue working throughout the pandemic while their less skilled counterparts were furloughed or otherwise unable to work (ONS 2021b).

Companies may also have used the lockdown period to upgrade and improve business systems, which might make them better prepared for a productivity-driven recovery. Investment data for the UK show that business investment in ICT equipment and other machinery and equipment has fared better during the pandemic than that for buildings and transport equipment. Investment in intellectual property products has also performed relatively well in the UK during the pandemic, suggesting that digital transformation is accompanied by increased investment in productivity-enhancing assets (ONS 2021f).

The overall productivity trend during the post-pandemic period will ultimately depend on whether such positive effects outweigh possible negative effects from the pandemic, in particular scarring effects on labour markets and business dynamics. A key risk is that the pandemic may have raised inequalities between particular occupational groups and places in terms of access to jobs and potential for productivity growth. This could be exacerbated by the displacement effects from new digital technologies. There could be a slowdown in the creation of new digital and other skills among workers not having gained on-the-job experience. This may cause an unbalanced or K-shaped recovery with large pockets of weak demand and slow investment across the economy.

A second risk to a recovery in productivity relates to the evolution of business dynamics during the post pandemic period. According to the ONS, business closures in the UK dropped over the course of 2020, but started to pick up again above previous years' average in Q4-20 and Q1-21 (ONS 2021d). The largest number of business closures occurred in the finance and insurance, real estate, and information and communication industries. Towards the end of 2020 and Q1-21, the number of new businesses created picked up substantially above the average of previous years, especially in retail, wholesale, and transportation and storage industries. These business dynamics will shape the extent to which resources (labour, capital, land) released from ailing firms will be absorbed by firms that are more productive.^12^ One risk is that firm births do not necessarily occur in the sectors that have shown high levels of productivity in the past (NIESR 2021). More broadly, recent research suggests that the business environment in the US has not been very conducive to dynamic market competition lately, even though European countries may have shown more competitive dynamics (Philippon 2019).

Finally, the large differences in within-industry productivity performance between countries also suggest that country-specific factors such as the response of innovation ecosystems to the opportunities for adoption of new technologies play an important role in explaining differences in productivity growth between countries during the next decade. Such differences in policy environment may also have a significant impact on the within-country regional distribution of a productivity revival.

## Appendix 1: Detailed industry quarterly productivity statistics for the UK, France and the US

Detailed quarterly industry productivity data on nominal and real value added and total hours worked are readily available for the UK from the ONS.^13^ The data is available for 66 industries, which is the level of detail that we use for the analysis throughout this paper. Quarterly detailed industry productivity data for France and the US are however not readily available, and a main contribution of this paper is indeed the development of these series (see Tables 4, 5, 6).

**Table 4:** Decomposition of within-industry productivity effects using three taxonomies, by quarter, France (% change). *Sources* “Appendix 1”. Based on INSEE (France)

	2020-Q1	2020-Q2	2020-Q3	2020-Q4	2021-Q1
Aggregate output per hour	− 1.5	6.3	− 3.5	1.3	0.0
Within-industry productivity	− 2.5	4.7	− 1.1	0.6	− 0.2
Static effect	1.1	2.0	− 2.2	0.8	0.3
Dynamic effect	− 0.1	− 0.4	− 0.2	− 0.1	0.0
Sectors					
Total within-industry effect	− 2.5	4.7	− 1.1	0.6	− 0.2
Manufacturing	− 0.4	0.0	0.2	0.2	0.1
Other industry^a^	− 0.3	0.0	0.0	− 0.1	− 0.1
Market services^b^	− 1.3	4.1	− 1.7	0.4	− 0.4
Hospitality and culture	− 0.3	0.5	− 0.3	0.2	0.0
Non-market services	− 0.2	0.1	0.7	− 0.1	0.1
Work-from-home (WFH) intensity					
Total within-industry effect	− 2.5	4.7	− 1.1	0.6	− 0.2
High WFH	0.1	1.3	− 1.3	0.5	− 0.3
Medium WFH	− 1.3	3.1	0.3	0.1	0.0
Low WFH	− 1.3	0.3	− 0.1	− 0.1	0.0
Digital intensity					
Total within-industry effect	− 2.5	4.7	− 1.1	0.6	− 0.2
Most digital intensive-using	− 1.2	2.6	− 0.4	0.1	− 0.6
Least digital intensive using	− 1.2	1.5	− 0.5	0.3	0.2
Digital producing	− 0.1	0.6	− 0.2	0.1	0.2

^a^Other industry includes agriculture, mining, utilities and construction

^b^Market services excludes Hospitality and culture as well as owner occupied dwellings

**Table 5:** Decomposition of within-industry productivity effects using three taxonomies by quarter, UK (% change). *Sources* “Appendix 1”. Based on ONS

	2020-Q1	2020-Q2	2020-Q3	2020-Q4	2021-Q1
Aggregate output per hour	− 0.9	− 3.1	8.5	− 4.2	0.5
Within-industry productivity	− 1.5	− 7.7	9.8	− 3.7	0.0
Static effect	0.7	3.9	− 1.8	− 0.2	1.0
Dynamic effect	− 0.1	0.7	0.4	− 0.3	− 0.4
Sectors					
Total within-industry effect	− 1.5	− 7.7	9.8	− 3.7	0.0
Manufacturing	0.0	− 0.6	1.4	− 0.5	0.0
Other industry^a^	− 0.3	− 0.6	1.5	− 0.7	0.4
Market services^b^	− 0.7	− 1.9	3.3	− 2.0	0.1
Hospitality and culture	− 0.2	− 2.1	1.9	− 1.0	0.6
Non-market services	− 0.4	− 2.6	1.7	0.5	− 1.1
Work-from-home (WFH) intensity					
Total within-industry effect	− 1.5	− 7.7	9.8	− 3.7	0.0
High WFH	− 0.6	− 1.8	2.0	− 0.6	− 0.6
Medium WFH	− 0.5	− 2.2	3.2	− 1.2	0.1
Low WFH	− 0.4	− 3.7	4.7	− 1.9	0.5
Digital intensity					
Total within-industry effect	− 1.5	− 7.7	9.8	− 3.7	0.0
Most digital intensive-using	− 0.7	− 1.8	3.7	− 2.2	0.2
Least digital intensive using	− 0.9	− 5.7	6.1	− 1.6	− 0.3
Digital producing	0.1	− 0.2	0.0	0.1	0.1

^a^Other industry includes agriculture, mining, utilities and construction

^b^Market services excludes hospitality and culture as well as owner occupied dwellings

**Table 6:** Decomposition of within-industry productivity effects using three taxonomies, by quarter, US (% change). *Sources* “Appendix 1”. Based on BEA and BLS

	2020-Q1	2020-Q2	2020-Q3	2020-Q4	2021-Q1
Aggregate output per hour	− 0.2	2.4	1.0	− 0.9	0.8
Within-industry productivity	− 0.3	− 0.3	2.6	− 0.7	1.0
Static effect	0.1	2.6	− 1.6	− 0.2	− 0.2
Dynamic effect	0.0	0.1	0.0	0.0	0.0
Sectors					
Total within-industry effect	− 0.3	− 0.3	2.6	− 0.7	1.0
Manufacturing	0.0	0.0	0.7	0.0	0.0
Other industry^a^	0.4	0.2	− 0.1	− 0.2	0.1
Market services^b^	− 0.4	0.3	1.2	− 0.4	1.0
Hospitality and culture	− 0.1	− 0.6	0.3	− 0.1	0.1
Non-market services	− 0.2	− 0.2	0.6	0.0	− 0.2
Work-from-home (WFH) intensity					
Total within-industry effect	− 0.3	− 0.3	2.6	− 0.7	1.0
High WFH	0.1	− 0.1	0.5	− 0.2	0.4
Medium WFH	− 0.4	0.4	1.3	− 0.2	0.4
Low WFH	0.0	− 0.7	0.8	− 0.2	0.2
Digital intensity					
Total within-industry effect	− 0.3	− 0.3	2.6	− 0.7	1.0
Most digital intensive-using	− 0.5	0.0	1.2	− 0.1	0.5
Least digital intensive using	0.1	− 0.9	1.3	− 0.6	0.1
Digital producing	0.0	0.5	0.1	0.0	0.4

^a^Other industry includes agriculture, mining, utilities and construction

^b^Market services excludes hospitality and culture as well as owner occupied dwellings

Data for France are mostly derived from annual and quarterly national accounts data provided by INSEE and detailed short-term business statistics provided by Eurostat. Our starting point is the quarterly national accounts data on nominal and real value added and total hours worked for 17 broad sectors. We then use more detailed annual national accounts data to break up those 17 broad sectors into 50 more detailed sectors for 1 year. To derive the quarterly movement of value added and hours worked for those 50 sectors we use short-term business statistics on output and labour input, sourced from Eurostat. Finally, we make sure that the quarterly movements sum up to the broad sectors derived from the quarterly national accounts data.

The quarterly productivity series for the US were derived from various datasets sourced from the BEA and BLS. The BLS maintains various productivity datasets, but the industry detail in the quarterly series is very limited. More importantly, non-market and non-farm output are excluded, hampering international comparability. Therefore our starting point is the GDP by industry data from the BEA. We then matched these detailed industry nominal and real value added data with labour input data. The labour input data by industry are sourced from the Current Employment Statistics (CES). However, these data are on an ‘hours paid’ basis (not on an 'hours worked' basis) and exclude various segments of the workforce such as the self-employed, agriculture and public sector workers. We use ratios of hours paid over hours worked from a number of broader sectors (or ‘parent industries’) to arrive at hours worked series at a detailed industry level. We then use unpublished employee/non-employee ratios derived from the CPS to arrive at total hours worked for all workers. Finally, we supplement the series with total hours worked measures on farm workers and public sector employees. In a final step, we make sure that the quarterly total economy total hours worked align with the quarterly sector totals from the BLS. This allows us to analyse the productivity movements of 48 detailed industries in the US economy.

When the goal is to tease out reallocation effects in productivity analysis, it is important to take out the value of owner-occupied housing. This is estimated in the compilation of GDP in the national accounts by assuming that home-owners pay market rents. However, as this value has no labour equivalent (at least not in a narrow ‘production boundary’ sense), the implied ratio of output to labour input (or, the level of productivity) of the real estate sector is strongly inflated when including owner-occupied housing. We therefore removed the value of owner occupied dwellings from nominal and real value added on a quarterly basis, thereby bringing the productivity levels of the real estate sector more in line with the market services aggregate productivity level.

## Appendix 2: Alternative aggregation methods

This paper uses a shift-share decomposition of aggregate productivity growth to distinguish pure or within-industry productivity effect from hours reallocation effects. An important advantage of the shift-share method, especially when looked at from a structural change perspective, is the ability to distinguish between the static and dynamic reallocation effects. Another important feature is the additivity of real output, so that aggregate output can simply be obtained by summing output across industries. However, as the changes in output prices differ across industries at a different pace, the shift-share decomposition is sensitive to the base year chosen. We discuss two alternative aggregation procedures that consider the price differences across industries—the Tornqvist aggregation and the Tang and Wang aggregation approach.

### The Tornqvist aggregation

In Eq. (1) of the main text, aggregate real value added is the sum of industry value added, assuming identical value added function across industries. The alternative Tornqvist aggregation procedure relaxes the assumption of an identical value-added function across industries by defining aggregate value added growth as a weighted average of industry value added growth rates, thereby sacrificing the additivity of industry output. In this approach, aggregate value added growth is a translog index of industry value added growth, allowing estimation of aggregate labour productivity growth:

4 $$\Delta \mathrm{ln} {y}_{t}^{*} =\sum {\overline{v} }_{i,t}\mathrm{ln} {Y}_{i,t} -\Delta \mathrm{ln} {H}_{t},$$

where $${\overline{v} }_{i,t}$$ is the share of industry i in aggregate nominal value added, averaged over the years t and t − 1. Note that $$\Delta \mathrm{ln} {y}_{t}^{*}$$ in (4) is not the same as the growth rate of value added per hour ($${y}_{t})$$ in (1), as the assumption of additivity imposed in Eq. (1) is relaxed in (4). Stiroh (2002) provides a useful decomposition of (4) into pure productivity and worker reallocation components. Defining aggregate hours growth as a translog index of industry hours growth (with the value added weights) and replacing for $$\Delta \mathrm{ln} {H}_{t}$$ in (4), we have

5 $$\Delta \mathrm{ln} {y}_{t}^{*} =\sum {\overline{v} }_{i,t}\mathrm{ln} {y}_{i,t} +{R}_{L,t},$$

where ${R}_{L,t}=\sum {\overline{v} }_{i,t}\cdot \Delta \mathrm{ln} {H}_{i,t}-\Delta \mathrm{ln} {H}_{t}$. In (5), the first term is the direct productivity effect. When the productivity of an industry improves, the aggregate productivity also improves in proportion to industry size (or the share in value added $v$). The second term, ${R}_{L}$, is a reallocation of working hours to sectors with relatively high productivity levels. It implies that aggregate productivity improves if industries with value added shares above employment shares experience employment growth. In other words, if workers move to sectors where relative levels of labour productivity are high, ${R}_{L}$ will be positive. In this approach, the worker reallocation can be easily obtained as a residual after subtracting the translog index of industry labour productivity growth from aggregate labour productivity growth obtained using production possibility frontier (Eq. 2). This approach also helps one identify the contribution of each individual industry to aggregate within or pure productivity effect, which is nothing but the share weighted sum of individual industry productivity growth.

In terms of the within-industry productivity effect, the difference between the shift-share and Tornqvist aggregation methods is primarily that the log difference in industry productivity is aggregated using industry output share according to Tornqvist whereas the shift-share method uses the industry employment shares in the previous period together with absolute differences in productivity.

### Tang and Wang decomposition

Tang and Wang (2004) introduced another alternative approach that addresses the sensitivity of aggregation to price differences between industries.^14^ In the shift-share and Tornqvist approaches, we indicated that the aggregate labour productivity is seen as the sum of within-industry productivity—contribution of individual industries—and the reallocation of workers across sectors. Tang and Wang (2004) suggests a third component, which is the rise of industry output price. Given that real GDP is the measured as nominal GDP divided by aggregate GDP price deflator, nominal GDP ($${Y}^{n})$$ is the product of real GDP ($$Y$$) and implicit GDP deflator ($${P}^{y})$$, i.e. ($${Y}_{t}^{n}={P}_{t}^{y}{Y}_{t})$$. Therefore, aggregate labour productivity is defined as $${y}_{t}={Y}_{t}^{n}/{{P}_{t}^{y}H}_{t}$$. Since the numerator, aggregate nominal GDP, can be obtained by summing across industries ($${Y}_{t}^{n}=\sum {Y}_{i,t}^{n})$$, aggregate labour productivity can be written as industry summation as:

6 $${y}_{t}=\frac{\sum {Y}_{i,t}^{n}}{{{P}_{t}^{y}H}_{t}}=\frac{\sum {Y}_{i,t}{P}_{i,t}^{y}}{{{P}_{t}^{y}H}_{t}}.$$

Dividing the numerator by H/H, we have

7 $${y}_{t}=\frac{\sum {Y}_{i,t}{P}_{i,t}^{y}{H}_{i,t}/{H}_{i,t}}{{{P}_{t}^{y}H}_{t}}=\frac{\sum {P}_{i,t}^{y}{H}_{i,t} {y}_{i,t}}{{{P}_{t}^{y}H}_{t}}=\sum {w}_{i,t} {y}_{i,t},$$

where ${w}_{i,t}=\left({P}_{i,t}^{y}/{P}_{t}^{y}\right) \cdot \left({L}_{i,t}/{H}_{t}\right)$, i.e., the product of relative output prices (the industry output price relative to the aggregate output price) and the industry employment share in the total economy. In other words, Tang and Wang's decomposition uses employment shares adjusted by output price to weight industry labour productivity to obtain aggregate labour productivity. The weight $w$ is equivalent to the revenue share of workers, or the share of the product of output prices and industry employment in the product of output prices and total economy employment. Transforming this into growth rate form,

8 $${\dot{y}}_{t}=\sum {v}_{i,t-1}{\dot{y}}_{i,t}+\sum {y}_{i,t-1}\Delta {w}_{i,t}+\sum {y}_{i,t-1}\Delta {w}_{i,t} {\dot{y}}_{i,t}.$$

As in (3) ${\dot{y}}_{t}= \left({y}_{i,t}/{y}_{i,t-1}\right)-1$ is the growth rate of labour productivity, and as in (4), $v$ is the nominal value added share of industry i in total GDP.

The first component is the product of labour productivity growth and nominal output share in the base year. This basically measures each industry's contribution to aggregate productivity growth, weighted by its relative size in the industry. Thus, as in the Tornqvist approach, the within-industry productivity effects in this approach is based on the growth rate of industry productivity weighted by industry value added share although Tang and Wang use base year weights, whereas the Tornqvist approach uses the average output share in the current and base year. An important feature of this decomposition is in its treatment of the second term, the reallocation effect.

The second term is the product of relative levels of labour productivity for any given industry in the initial period and the change in employment share, where the latter is adjusted for relative output prices. This indicates whether employment is shifting to sectors where the relative level of productivity is high—similar to the static effect in the shift-share analysis. However, productivity levels are expressed in relative terms, and the employment shares are expressed in nominal terms. Therefore, the change in the relative size of an industry can happen either because of a quantitative shifts in employment or because of a change in output prices (or a combination of the two). The last term is the second term times labour productivity growth, and hence an interaction term, which measures the so-called Baumol effect—i.e., whether resources move from low growth to high growth sectors—similar to the dynamic effect in the shift-share analysis. In the shift-share method the dynamic effect is the product of change in employment share and change in productivity, whereas here it is the product of change in employment (adjusted for relative prices) share, productivity growth and the previous period relative labour productivity level.

A disadvantage of the Tang and Wang method is that it is difficult to distinguish between the pure effect of a shift in employment across sectors and the change in relative prices, as it uses both measures as weights. A decline in employment share can be offset by a rise in relative output prices (De Avillez 2012).^15^ For instance, as noted by Reinsdorf (2015), an increase in the prices of products produced in a domestic industry due to a fall in imports of those products (or due to any sort of import restriction) may result in counting that industry's contribution positively to aggregate productivity growth. This is not necessarily a labour reallocation effect, but a reflection of the windfall gain accrued to that industry due to the apparent decline in imports. Reinsdorf (2015), argues that treating an increase in the output price of an industry as a positive contribution to aggregate productivity growth is inconsistent with definition of productivity growth.^16^ Thus, as this approach interprets a rise in industry output price as a rise in productivity, it is not a measure of productivity contribution in the traditional sense, rather an estimate of economic value generated by industries. For instance, in this approach, it is likely that high-tech industries with massive increases in real output and productivity contribute negatively to aggregate productivity growth if they see rapid declines in output prices owing to technology improvements. In contrast, industries with falling productivity and rising prices can make positive contributions.^17^

Table 7 compares the differences in the industry reallocation effects according to the shift-share, Tornqvist and Tang and Wang methods for the UK. First, we find that the reallocation effects in 2020 are indeed extremely large compared to a normal year like 2019, especially for Q2-20 and Q3-20. Second, we find that the Tang and Wang method tends to provide somewhat larger reallocation effects than the other two methods, which especially impacted Q2-20 and Q3-20 (see Table 7).

**Table 7:** Industry labour reallocation effects under alternative aggregation approaches (percentages): UK (%, quarter over quarter)

	2019				2020			
	Q1 (%)	Q2 (%)	Q3 (%)	Q4 (%)	Q1 (%)	Q2 (%)	Q3 (%)	Q4 (%)
Shift-share	0.1	0.1	0.1	− 0.5	0.6	4.6	− 1.4	− 0.4
Tornqvist	0.2	0.1	0.1	− 0.4	0.7	4.2	− 1.4	− 0.4
Tang and Wang	0.1	0.3	0.1	− 0.5	0.7	6.3	− 4.2	− 1.0

Real estate sector is excluded from the aggregation

Since our focus is on the pure or within-industry productivity effects, it may be noted that in all these three approaches, the within effect is a weighted aggregate of industry productivity growth. However, the weights differ—hours (shift-share) or output share (Tang and Wang approach) in the previous period, or a two period average of output shares (Tornqvist). In this paper, we opt for the shift-share approach, which weighs industry productivity growth rates by their base year level of total hours. We acknowledge that the pure effect we consider in the paper takes care of only the movement of workers between sectors and not of measurement issues or price differences between sectors as discussed above.

## Footnotes

1 https://www.conference-board.org/data/economydatabase/, April 2021.

2 https://www.conference-board.org/data/economydatabase/, April 2021.

3 Krugman’s phrase was “Productivity isn’t everything, but in the long run it is almost everything. A country's ability to improve its standard of living over time depends almost entirely on its ability to raise its output per worker.”.

4 Fernald (2014) and ONS (2021b).

5 For other analysis along those lines, see ONS (2021b) for the UK and Blit et al. (2020) and Wang (2021) for Canada.

6 In Q1-2021 the within-industry productivity growth over Q4-2020 was − 0.2% in France, 0% in the UK and 1% in the US (Appendix Tables 4, 5, 6).

7 The underlying assumptions include separability of gross output production function in value added for each industry and same value added function across all industries.

8 The quantitative impact of different levels of aggregation is demonstrated for the UK by ONS (2021b, Fig. 4). Their decomposition using five sectors: agriculture, mining and utilities, manufacturing, construction, financial services, and all non-financial services, shows smaller reallocation effects compared to their sectional aggregation containing 17 industries and divisional aggregation consisting of nearly 90 detailed industry groups. The results show that the detailed industry breakdown provides larger reallocation effects, although there can also be offsetting effects between industries.

9 Comparison of market services and non-market services results with the US is somewhat flawed as the “non-market services sector” includes health and education which to a large extent are private sector activities in the US.

10 The projections by The Conference Board are based on a supply-side growth accounting model that estimates the contributions of the use of factor inputs—labour and capital—and total factor productivity growth to the growth of real gross domestic product (GDP). While labour input growth rates are estimated using data on demographic changes and participation rates—including an estimation to adjust for the change in the composition (or quality) of the workforce—capital input and total factor productivity growth are econometrically estimated using a wide range of related variables during past periods. The obtained trend growth rates for the first projection period (currently 2021–2023) are adjusted for possible deviations between actual and potential output in the short run (Erumban and de Vries 2018).

11 The displacement effects reflect the shift in the task content from labor in favor of capital and improving productivity due to automation, whereas the reinstatement effect is the offsetting effect of automation by creating new tasks (Acemoglu and Restrepo 2019).

12 See also OECD (2021b).
